# The Best Dentistry Professional Visual Acuity Measured under Simulated Clinical Conditions Provides Keplerian Magnification Loupe: A Cross-Sectional Study

**DOI:** 10.3390/dj9060069

**Published:** 2021-06-11

**Authors:** Iris Urlic, Josip Pavan, Zeljko Verzak, Zoran Karlovic, Dubravka Negovetic Vranic

**Affiliations:** 1 Department of Ophthalmology, Clinical Hospital Dubrava, Av.GojkaŠuška6, 10000 Zagreb, Croatia; josip.pavan@kbd.hr; 2 Department of Pediatric and Preventive Dentistry, School of Dental Medicine, University of Zagreb, Gundulićeva 5, 10000 Zagreb, Croatia; verzak@sfzg.hr (Z.V.); dnegovetic@sfzg.hr (D.N.V.); 3 Department of Endodontics and Restorative Dentistry, School of Dental Medicine, University of Zagreb, Gundulićeva 5, 10000 Zagreb, Croatia; karlovic@sfzg.hr

**Keywords:** visual acuity, miniaturized Snellen optotype, Galilean and Keplerian telescope optical system in dentistry

## Abstract

Visual acuity plays an important role in dentists’ vision in their daily clinical routine. This study aimed to determine dental students’ visual acuity without optical aids and when using magnification devices in simulated clinical conditions. The participants were forty-six students at the School of Dental Medicine with a visual acuity of 1.0 in decimal values or 100% in percentage. The central visual acuity was tested using a miniature Snellen eye chart placed in the molar cavity of a dental phantom, in simulated clinical conditions under five different settings (natural visual acuity, by applying head magnifying glasses x1,5 and binocular magnifying devices using Galileo’s x2,5/350 mm, Keplerx3,3/450 mm and Keplerx4,5/350 mm optical system). The Wilcoxon Signed Rank test shows that the distribution of measurements of the visual acuity undertaken by the application of magnifying devices (VNL, VGA2,5, VKP3,3, VKP4,5) contained higher values of visual acuity than those received by the use of natural vision (VSC) (*p* < 0.001 for the comparison to the VNL, VGA2,5, VKP3,3 and VKP4,5 groups). The highest and statistically most significant increase in visual acuity is achieved using the Keplerian telescope x4.5/350 mm. The application of magnifying devices provided dentistry professionals with better visual acuity, improving detail detection in an oral cavity during dental procedures by magnifying the oral structure. The use of magnification devices means much more precise work, decreases the operating time, improves posture and reduces muscle pain in the shoulder during dental treatment.

## 1. Introduction

By using magnifying aids, a dentist achieves ergonomic, musculoskeletal and optical benefits, and the magnification compensates for the weakening of eye accommodation, which occurs after the age of forty (presbyopia). The use of telescope magnification devices proved to improve the diagnosis and treatments in dental medicine [1,2]. Magnification in dentistry upgrades soft and hard tissue evaluation, calculus and periodontal pocket detection, restorative evaluation and radiological interpretation [3,4].

Highly demanding eye-hand coordination and tactile perception require the highest visual function, three-dimensional image creation, stereo vision and object depth perception as well as other psychological and neurological qualities [3].

Magnification is achieved by a system of lenses used in the Galileo and Kepler optical systems. The Galilean telescope has a diverging and the Keplerian telescope a converging lens eyepiece [5]. The loupe’s ergonomic factors are the declination angle, working distance and frame size [6].

A study that is examining the visual acuity of dentists with age proved a wide variability in near visual acuity and a significant improvement in visual acuity within the dental workspace by 100–379% when using Galileo’s x2,5 and Kepler’s x4,3 magnifying glass, regardless of age [7]. Kepler’s magnifier x4,3 achieved the highest visual acuity in the clinical work of dentists due to the highest magnification, but also due to the absence of chromatic and optical aberrations [8]. Dentists under the age of 40 statistically have significantly greater visual acuity than older dentists. Dentists under the age of 40 can significantly improve their visual acuity by reducing the eye–object distance or by using magnifying glasses [9]. The use of magnification aids does not weaken the eye. After wearing loupes for a period, the user becomes accustomed to seeing more detail than is apparent with natural vision [10].

The most commonly used proximity visual test of the British Faculty of Ophthalmology [11] is not sensitive enough to examine visual acuity in the dentist’s workplace, and a standardized, generally accepted proximity visual acuity test in dentistry with magnifications, which would meet optical parameters, is not yet available [8].

The miniaturization of Snellen’s optotype that was carried out for this research enabled the study to examine how properly fitted magnification loupes improve visual acuity at close range, therefore producing higher-quality dentistry.

## 2. Materials and Methods

### 2.1. Participants

The study was carried out in the Dentistry Pre-clinic of the School of Dental Medicine, University in Zagreb from September to December 2015. The participants were students at the School of Dental Medicine, University of Zagreb (N = 46) who voluntarily agreed to participate in the study. The participants were of both genders, aged between 20 and 25 years. Before being included in the study, their eye status was examined by an ophthalmologist in an ophthalmology practice. The inclusion criteria for participants were a central visual acuity of 1.0 in decimal values examined using a Snellen visual chart, a near visual acuity of 1.0 examined using Jäger tables, regular ocular movement and convergence, regular pupil reaction and an oval or round shaped optic nerve head.

### 2.2. Miniature Snellen Visual Test

**Figure 1:**
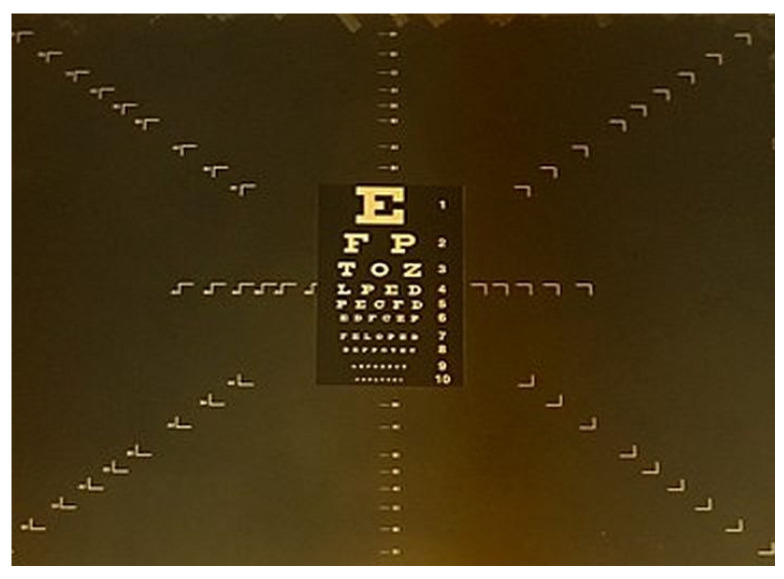
Miniaturized visual Snellen optotype (under magnification ×4) by courtesy of the Croatian State Archive.

The visual acuity was examined by means of a miniature visual test invented for this study in cooperation with the Croatian State Archives, Central Photo Laboratory. A sample of an A4 Snellen optotype was created in high resolution, printed and copied onto a 35-millimeter B/W microfilm. A microfilm camera, Zeutscheu Documator, was used to reduce the high-resolution A4 Snellen chart to the highest possible reduction of 28,5x compared to the initial size of the optotype. The size of the miniaturized Snellen chart for the near visual acuity examination of dentists was 5.2 × 2.8 mm and the optotype dimensions ranged from 0.05 mm to a maximum of 0.6 mm (Figure 1).

### 2.3. Dentistry Professional Visual Acuity with Magnification Telescopes

**Figure 2:**
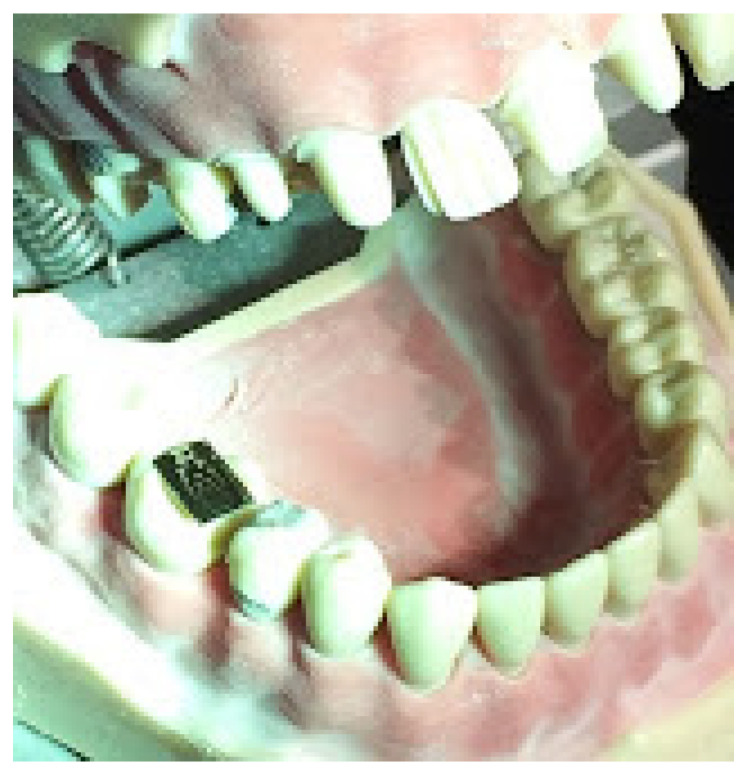
Miniaturized visual Snellen chart placed in the tooth cavity of a dental phantom.

**Figure 3:**
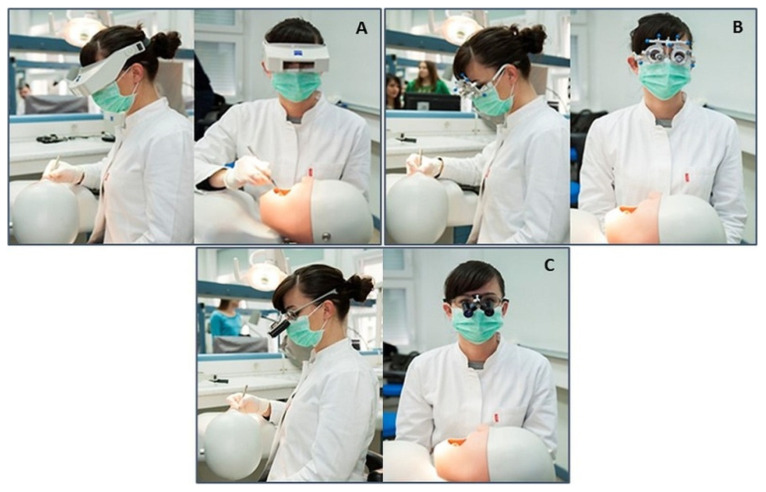
Examining the dentistry visual acuity under simulated clinical conditions by applying (**A**) head magnifying glasses, (**B**) a Galilean telescope, (**C**) and a Keplerian telescope.

The participants were examined in a working zone lit by a 60 W surgery lamp parallel to the participants’ visual axis, sitting in a dental chair with a maximum head deflection of 25° forward in an upright position. Their feet were fully placed on the floor, their knees were placed below and their elbows level with the dental phantom. The examination room was illuminated by sunlight and artificial light ranging from 250 to 500 lux, measured using a luxmeter. The miniature visual chart was fixed in the dental phantom’s molar cavity (Figure 2). The visual acuity was registered by decimal graduation between 0.1 to 1.0 as the smallest optotype of the miniaturized Snellen optotype that an examinee managed to read at a working distance without correction (VSC), by application of x1.5 head magnifying glasses (VNL), using Galileo’s x2,5/350 mm binocular magnifying devices (VGA2,5), Kepler x3,3/450 mm optical magnification system (VKP3,3) and a x4,5/350 mm optical system (VKP4,5) (Figure 3). The study was approved under No. 05-PA-26-6/2015 by the Ethics Committees of the School of Dental Medicine, University of Zagreb.

### 2.4. Statistical Analysis

Prior to performing the differential analysis, the normality of the data’s distribution was tested. The Shapiro–Wilk test rejected the null hypothesis of the data’s normality (*p* < 0.05). As the data were not normally distributed, nonparametric statistical tests were used for their analysis.

Data on visual acuity during the use of five different optical systems were collected from the same subjects and analyzed using the following non-parametric tests for repeated measurements: the Friedman test and the Wilcoxon Signed Rank test. The Friedman test examined whether there was a statistically significant difference in visual acuity between the different optical systems. In order to find out between which pairs of systems significant deviations in visual acuity were recorded, the Wilcoxon’s Signed Rank test was performed after the Friedman test. The analysis was performed using the SAS System software package (SAS Institute Inc., Cary, NC, USA).

## 3. Results

### 3.1. Standard Deviation of Dentistry Professional Visual Acuity

Statistical analysis included 46 subjects, 25 females and 21 males, with an average age of 21.8 years. Visual acuity was examined at close range within each individual group and the following were compared: the control group with each individual group corrected by a magnifying aid; visual acuity in the vicinity of the Kepler/Galileo systems; visual acuity in the vicinity of the Keplerian optical systems.

The mean value (standard deviation) of visual acuity using natural vision without magnifying aids at a distance of 300 to 400 mm (VSC) was 0.411 (0.074); using head magnification glasses x1,5 (VNL), 0.504 (0.076); using a Galilean magnifying telescope x2,5 at a distance of 350 mm (VGA2,5), 0.517 (0.077); using a Kepler optical system x3,3 at a distance of 450 mm (VKP3,3), 0.541 (0.086); and when using Kepler x4.5 at a distance of 350 mm (VKP4,5) the average value (standard deviation) of the recorded visual acuity measurements was 0.646 (0.081) (Table 1).

### 3.2. The Friedman Test

Consistent with the results of the descriptive analysis, the Friedman test indicated the existence of statistically significant differences in the distribution of the visual acuity measurements between the different optical systems (*p* < 0.001).

### 3.3. The Wilcoxon Signed Rank Test

A comparison of a VNL with a VGA 2,5, VKP 3,3 and VKP4,5 indicated a statistically significant difference in the distribution of the visual acuity measurements in relation to Kepler’s magnifying glass x3,3 (VKP 3,3; *p* = 0.014) and Kepler’s magnifying glass x4.,5 (VKP4,5; *p* < 0.001), but not in relation to the Galilean magnifying glass x2,5 (VGA 2,5; *p* = 0.288). When using Kepler’s magnifiers, higher values of visual acuity were generally recorded. The Wilcoxon Signed rank test indicated a statistically significant difference between the distributions of visual acuity (VGA 2,5) in relation to Kepler’s magnifying glass x4,5 (VKP4,5; *p* < 0.001), but not in relation to Kepler’s magnifying glass x3,3 (VKP 3,3; *p* = 0.064). Compared to the Galilean magnifying glass x2.5 (VGA 2,5), Kepler’s magnifying glass x4,5 (VKP4,5) generally recorded higher values of visual acuity. A comparison of Kepler’s magnifiers indicated that the distribution of the measured visual acuity values after using the Kepler magnifying glass x4,5 (VKP4,5) generally contained higher visual acuity values compared to the distribution of measured values after using the Kepler magnifying glass x3,3 (VKP 3,3) (Wilcoxon Signed Rank test; *p* < 0.001). The results of the Wilcoxon Signed Rank test concluded that the distributions of visual acuity measurements when using magnifying aids (groups VNL, VGA 2,5, VKP 3,3 and VKP4,5) generally contained higher values of measured visual acuity compared to the use of natural vision without magnifying aids (VSC) (*p* < 0.001 for comparison with VNL, VGA 2,5, VKP 3,3 and VKP4,5 groups) (Table 2).

## 4. Discussion

The range of visual acuities close to the normal eye status of subjects who do not use optical aids for eye correction is from 0.3 to 0.6 in decimal values. Statistically significant near visual acuity compensation relative to visual acuity without optical aids is achieved using a head magnifier x1,5, a Galileo telescope x2,5 and a Kepler system x3,3, and the range of near visual acuity is from 0.4 to 0.7. There was no statistically significant difference between the visual acuity of head magnifier x1,5, Galileo x2,5 and Kepler x3,3. This study shows that it is justified to start using the Galileo optical systems x2,5 at a working distance of 350 mm for dental students, dental technicians and dentists who are beginning to adapt to magnification [12]. A study examining the use of magnifications at the New Zealand School of Dentistry found that out of 285 first-year students, 23% used a magnification of up to 48% in their final year of study. In addition, 72% of professors use magnifiers, with the most common magnification of x2.5, and half of them use magnifiers with an added light bulb [13]. Eichenberger et al. evaluated the self-assessed near visual acuity of sixty-nine dentists in a private practice in Switzerland as well as their experience with magnification devices. The study showed that many dentists were not aware of their individual or age-related visual deficiencies that can be compensated with telescope systems and should be used early enough to compensate for those vision irregularities [14].

With the Keplerian telescope x4,5, a minimum value of visual acuity of 0.5 is achieved and a maximum of 0.8. A statistically significant increase in visual acuity is achieved by increasing the magnification, changing the Galileo x2,5 to Kepler’s optical system x4,5 and by increasing the magnification within the Kepler x3,3 to x4,5.

Kepler’s x4.5/350 mm magnifier achieves a maximum visual acuity that is not achieved with any other magnifier used in this study. A study conducted by Wajngarten examined dental students’ visual acuity and forward head posture when using telescope systems in the operating field. The Galilean and Keplerian magnification systems provided the best visual acuity and the lowest angulation of the operator’s neck in comparison to the naked eye, simple loupe and operating microscope [15]. The visual threshold was reached with the Keplerian telescope by dentists under the age of 40, both with and without coaxial light, and dentists under the age of 40 identified a 0.05 mm structure within the root canal [16].

It was observed that 100% visual acuity or 1.0 by the Snellen optotype was not achieved even with the best correction, despite the fact that the study was performed in simulated conditions with young subjects, without refraction error or presbyopia. The use of turbine and the effect of aerosol on blurred vision were not taken into consideration.

This study includes emetropic (normovision) students without refractive anomalies (myopia, hyperopia). By involving dental students, we excluded dentists over the age of forty, presbyopic participants. A future step for this study is to examine the visual acuity of myopic, hyperopic and presbyopic dentists with and without optical aids.

## 5. Conclusions

The major optical goal for central visual acuity by using telescopes is to magnify the image in the dental visible path. Visual performance increases with the application of head magnifying glasses and binocular magnifying devices. The highest increase in visual acuity is achieved using the Keplerian telescope x4.5/350 mm. The Galilean telescope is small and lightweight but the Keplerian telescope, in comparison with Galileo’s, allows for a higher magnification, greater depth of field, wider field of view and greater focal length.

## Figures and Tables

**Table 1:** Descriptive indicators of visual acuity distribution *.

Optical System	N	Mean	Std Dev	CV (%)	Median	Q1	Q3	Min	Max
VSC	46	0.411	0.074	17.9	0.4	0.4	0.5	0.3	0.6
VNL	46	0.504	0.076	15.0	0.5	0.5	0.6	0.4	0.7
VGA 2,5	46	0.517	0.077	14.9	0.5	0.5	0.6	0.4	0.7
VKP 3,3	46	0.541	0.086	15.9	0.6	0.5	0.6	0.4	0.7
VKP4,5	46	0.646	0.081	12.5	0.6	0.6	0.7	0.5	0.8

* N, sample size; Mean, arithmetic mean; Std Dev, standard deviation; CV, variation coefficient; Q1, 1st quartile; Q3, 3rd quartile; Min, minimum; Max, maximum.

**Table 2:** Wilcoxon Signed Rank test results to compare visual acuity recorded when using different optical systems *.

Optical System Comparison	Test Statistics W	*p*-Value
VNL vs. VSC	390,0	<0.001
VGA 2,5 vs. VSC	403,5	<0.001
VKP 3,3 vs. VSC	423,0	<0.001
VKP4,5 vs. VSC	517,5	<0.001
VGA 2,5 vs. VNL	22,5	0.288
VKP 3,3 vs. VNL	103,5	0.014
VKP4,5 vs. VNL	473,0	<0.001
VKP 3,3 vs. VGA 2,5	55,0	0.064
VKP4,5 vs. VGA 2,5	480,5	<0.001
VKP4,5 vs. VKP 3,3	430,5	<0.001

* VSC, visual acuity without correction; VNL, visual acuity with the application of x1.5 head magnifying glasses; VGA2,5, visual acuity with the Galileo x2.5/350 mm magnifying device; VKP3,3, visual acuity with the Kepler x3,3/450 mm optical system; VKP4,5, visual acuity with the Kepler x4,5/350 mm optical system.

## Data Availability

The data presented in this study are available on request from corresponding author.
